# Interleukin-10 inhibits osteoclastogenesis by reducing NFATc1 expression and preventing its translocation to the nucleus

**DOI:** 10.1186/1471-2121-8-4

**Published:** 2007-01-19

**Authors:** Kathryn E Evans, Simon W Fox

**Affiliations:** 1 Ecotoxicology and Stress Biology Research Group, School of Biological Science, University of Plymouth, UK

## Abstract

**Background:**

IL-10 has a potent inhibitory effect on osteoclastogenesis. In vitro and in vivo studies confirm the importance of this cytokine in bone metabolism, for instance IL-10-deficient mice develop the hallmarks of osteoporosis. Although it is known that IL-10 directly inhibits osteoclastogenesis at an early stage, preventing differentiation of osteoclast progenitors to preosteoclasts, the precise mechanism of its action is not yet clear. Several major pathways regulate osteoclastogenesis, with key signalling genes such as p38, TRAF6, NF-κB and NFATc1 well established as playing vital roles. We have looked at gene expression in eleven of these genes using real-time quantitative PCR on RNA extracted from RANKL-treated RAW264.7 monocytes.

**Results:**

There was no downregulation by IL-10 of DAP12, FcγRIIB, c-jun, RANK, TRAF6, p38, NF-κB, Gab2, Pim-1, or c-Fos at the mRNA level. However, we found that IL-10 significantly reduces RANKL-induced NFATc1 expression. NFATc1 is transcribed from two alternative promoters in *Mus musculus *and, interestingly, only the variant transcribed from promoter P1 and beginning with exon 1 was downregulated by IL-10 (isoform 1). In addition, immunofluorescence studies showed that IL-10 reduces NFATc1 levels in RANKL-treated precursors and suppresses nuclear translocation. The inhibitory effect of IL-10 on tartrate-resistant acid phosphatase-positive cell number and NFATc1 mRNA expression was reversed by the protein kinase C agonist phorbol myristate acetate, providing evidence that interleukin-10 disrupts NFATc1 activity through its effect on Ca^2+ ^mobilisation.

**Conclusion:**

IL-10 acts directly on mononuclear precursors to inhibit NFATc1 expression and nuclear translocation, and we provide evidence that the mechanism may involve disruption of Ca^2+ ^mobilisation. We detected downregulation only of the NFATc1 isoform 1 transcribed from promoter P1. This is the first report indicating that one of the ways in which IL-10 directly inhibits osteoclastogenesis is by suppressing NFATc1 activity.

## Background

Osteoclasts are terminally differentiated TRAP-positive cells derived from monocyte-macrophage lineage precursors and are responsible for bone resorption. The process of osteoclast formation includes the proliferation and the differentiation of osteoclast progenitors into mononuclear preosteoclasts and the fusion of preosteoclasts into multinucleated osteoclasts. Excessive osteoclast activity plays a role in the development of several debilitating disorders such as osteoporosis, rheumatoid arthritis, and osteolytic metastases, and so it is vital that osteoclast development and function is properly regulated.

Some of the signalling pathways that contribute to osteoclast differentiation have started to emerge. Signalling by RANKL, an osteoblast-expressed member of the tumour necrosis factor superfamily, is essential for terminal differentiation of monocytes into osteoclasts. Resorptive stimuli initiate osteoclast formation by promoting the expression of osteoblastic RANKL, which binds to its receptor RANK. Binding of RANKL to RANK recruits several signalling intermediates including NF-κB, which is redistributed to the nucleus by TRAF family intermediates [1], and NFATc1, which induce the transcription of genes involved in osteoclast differentiation. NFATc1 is critical to osteoclast formation [2,3].

Negative regulators of RANKL signalling exist, including osteoprotegerin, a soluble decoy receptor for RANKL, and IFN-β, a negative regulator of transcription factor c-Fos expression [4]. Several cytokines, including IFN-γ, IL-4, and another tumour necrosis factor family member, TRAIL, interfere with the ability of RANKL to induce osteoclast differentiation. There is rapid degradation of TRAF6 abolishing signalling in the case of IFN-γ [5]. In the case of IL-4, signal transducer and activator of transcription 6 appears to be a key upstream regulatory molecule [6-9]. In the case of TRAIL, the mechanism is related to inhibition of the p38/MAPK pathway via reduced p38/MAPK phosphorylation [10].

IL-10 is a potent anti-inflammatory cytokine that suppresses both immunoproliferative and inflammatory responses. It is produced primarily from T cells and activated macrophages and acts on the macrophage lineage. Animal studies implicate IL-10 in the development and progression of arthritis [11-14] and chronic colitis [15,16]. IL-10 has a critical role in the in vivo regulation of pro-inflammatory cytokine levels [13,14,17]. It functions in a negative feedback loop, in which it suppresses the release of inflammatory cytokines and dampens the acute inflammatory response.

IL-10 also has potent inhibitory effects on osteoclastogenesis [18], but the molecular basis of its action is poorly understood. IL-10 inhibits the early stages of osteoclastogenesis, preventing differentiation of osteoclast progenitors to preosteoclasts. It has been shown to mediate this action through both direct and indirect actions. IL-10 indirectly inhibits bone resorption by upregulating osteoprotegerin expression while downregulating expression of RANKL and macrophage colony stimulating factor [19]. IL-10 also directly inhibits osteoclast formation [20]. It was found that the inhibitory effect of IL-10 on osteoclast formation is mediated through a direct action on osteoclast precursors [20,21]. Furthermore, enhanced osteoclastogenesis has been observed in cultures of bone marrow macrophages deficient in IL-10 production [22]. However, the molecular mechanism mediating this direct effect is unknown.

IL-10-deficient mice are now available and results from these mice confirm the importance of this cytokine in bone metabolism. They have been shown to develop the hallmarks of osteoporosis, namely, reduced bone mass, increased mechanical fragility, and suppressed bone formation [23]. IL-10 is an important endogenous suppressor of infection-stimulated bone resorption in vivo, and it appears that the anti-osteoclastic mechanisms induced by IL-10 on bone are more effective than those mediated by IL-4 [24]. IL-10-deficient mice exhibit significantly higher levels of periodontal alveolar bone loss than a matched control group [25]. This increased alveolar bone loss appears to be a late onset condition, and analysis of serum levels of type I collagen C-telopeptide suggests that lack of IL-10 may have a direct effect on bone homeostasis [26]. Clinical studies back up the theoretical importance of this work: an IL-10 promoter gene polymorphism has been found that is associated with reduced bone mineral density and predisposes women to osteoporosis at the lumbar spine [27].

In this paper we set out to learn more about how interleukin-10 directly inhibits the process of osteoclastogenesis by looking at its effects on selected gene expression in RAW264.7, a murine monocytic cell line.

## Results

### IL-10 suppresses osteoclast formation

**Figure 1:**
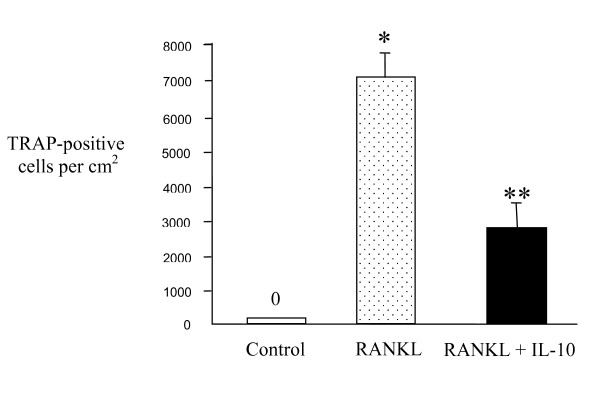
**Osteoclast formation is suppressed by IL-10**. RAW264.7 cells were incubated with RANKL or RANKL plus IL-10 for 5 days. All cytokine concentrations were 50 ng/ml. Cells were fixed and stained for TRAP and the number of TRAP-positive mono and multinuclear osteoclasts quantified. Results are expressed as the mean ± SEM for each group from three separate experiments. IL-10 significantly suppressed osteoclast formation. * p < 0.05 versus control, ** p < 0.05 versus RANKL-treated group.

Three groups of RAW264.7 cells were set up – an untreated control group, a group treated with RANKL, and a final group treated with RANKL plus IL-10. As shown previously, after five days numerous mono and multinuclear TRAP-positive cells were seen in the RANKL-treated cultures. The percentage of TRAP-stained cells in each group was determined by light microscopy and it was found that IL-10 significantly inhibits osteoclast formation but does not abolish it completely (Figure 1). These results indicate that IL-10 acts directly on monocytes to inhibit osteoclast differentiation.

### NFATc1 is downregulated by IL-10

**Figure 2:**
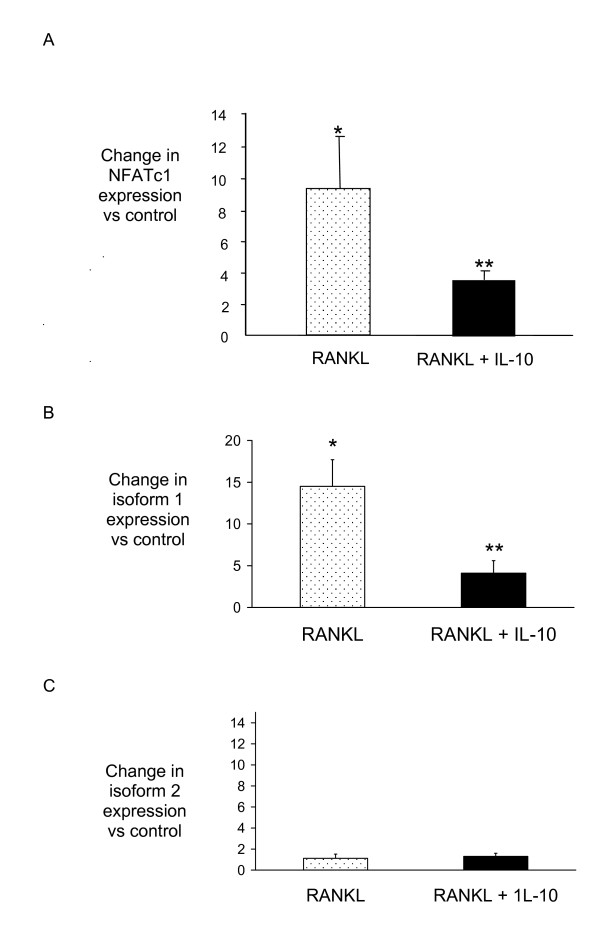
**NFATc1 expression is suppressed by IL-10**. RAW264.7 cells were incubated with cytokines (RANKL 50 ng/ml and IL-10 50 ng/ml) for 2 days and total RNA extracted. NFATc1 expression was assessed using quantitative PCR using specific primers recognising both isoforms (A) isoform 1 (B) or isoform 2 (C). Results are expressed as the mean ± SEM for each group from three separate experiments normalised for β-actin. * p < 0.05 versus control, ** p < 0.05 versus RANKL-treated group.

RANKL was found to induce the expression of NFATc1 mRNA approximately 8-fold, and this was significantly suppressed by co-incubation with IL-10 (Figure 2). This is in keeping with the direct action of IL-10 on osteoclast formation. NFATc1 expression was not however completely abolished. Isoforms of NFATc1 are known to exist in both humans [28] and mice [29]. These isoforms are transcribed from two separate promoters, which can be arbitrarily designated P1 and P2. Transcripts beginning from P1 start with exon 1 (isoform 1) and are longer than those beginning from P2 and starting with exon 2 (isoform 2). It is possible to design primers to differentiate between these variants and we did this. We found that RAW264.7 cells express both NFATc1 isoforms (isoform 1: 7.5 × 10^3 ^copies, isoform 2: 3.4 × 10^2 ^copies) and RANKL induced a significant 14.5-fold increase in isoform 1 expression, which IL-10 suppressed (Figure 2B). In contrast RANKL and IL-10 had little effect on the expression of isoform 2 (Figure 2C). Therefore, it appears that IL-10 may suppress osteoclastogenesis by blunting expression of NFATc1 isoform 1 rather than isoform 2. Because NFATc1 is central to osteoclast formation, any change in its expression would be expected to have a major effect on downstream gene expression.

### Expression of other osteoclast signalling genes is unaffected

None of the other osteoclast signalling genes tested – RANK, TRAF6, p38, NF-κB, c-Fos, c-jun, FcγRIIB, DAP12, Gab2, and Pim1 – were upregulated as strongly as NFATc1 by RANKL, and none of them were downregulated by IL-10 (data not shown). The key signalling factors known to trigger NFATc1 expression are TRAF6 and c-Fos [2], and so the lack of response of these genes in our experiments suggests that the effect of IL-10 on monocytes is not mediated through these pathways, in which case it must be mediated through an alternative mechanism.

### IL-10 alters cellular localisation of NFATc1

**Figure 3:**
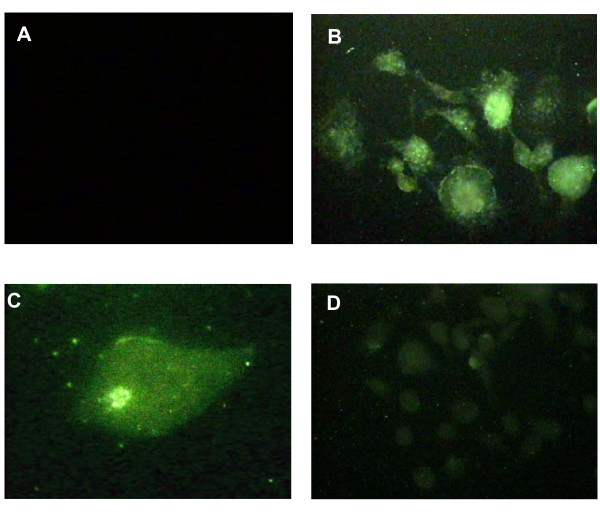
**IL-10 reduces NFATc1 immunostaining intensity and nuclear translocation**. NFATc1 staining was noted in the cytoplasm and nuclei of RANKL treated RAW264.7 cells (**B **and **C**), whereas only weak cytoplasmic staining was observed in RANKL + IL-10 treated cells (**D**.) No staining was noted in control cultures (**A**). Photographs taken at a magnification of × 400 A, B, D and ×1000 C.

NFATc1 is the most strongly induced transcription factor gene following RANKL stimulation [2,3]. RANKL also evokes Ca^2+ ^oscillations that lead to calcineurin-mediated activation of NFATc1. In resting T cells NFATc1 is restricted to the cytoplasm. Following T-cell activation, a sustained increase in intracellular calcium activates the phosphatase calcineurin. The activated calcineurin dephosphorylates NFATc1 and leads to increased nuclear accumulation. Therefore it was of interest to determine the cellular location of NFATc1 in the RANKL- and IL-10-treated monocytes. In keeping with our qPCR results, RANKL-treated cells showed intense cytoplasmic staining and nuclear accumulation was also noted (Figure 3). Similarly, again in keeping with our qPCR results, IL-10 reduced the intensity of cytoplasmic staining and little nuclear accumulation was observed in these cultures. This correlates with NFATc1 being highly active in the RANKL-treated cells and less active in the presence of IL-10.

### IL-10 may disrupt NFATc1 activity through its effect on Ca^2+ ^mobilisation

**Figure 4:**
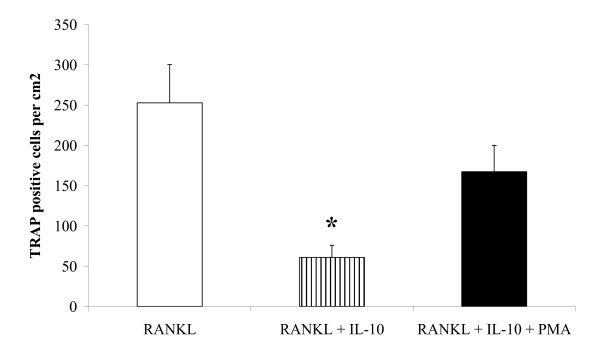
**PMA inhibits the suppressive effect of IL-10 on osteoclast formation**. RAW264.7 cells were incubated with RANKL, IL-10 or PMA for 3 days. Cultures were washed to remove PMA and then incubated for a further two days with RANKL or RANKL + IL-10. Cells were fixed and stained for TRAP and the number of TRAP-positive mono and multinuclear osteoclasts quantified. IL-10 suppressed the number of TRAP positive cells and this was prevented by PMA. Results are expressed as the mean ± SEM for each group from three separate experiments n = 9. * p < 0.05 versus all other groups.

PKC plays a critical role in T cell receptor-induced NFAT activation [30]. Deficiency of PKC primarily abrogates NFAT transactivation. This NFAT defect appears to be secondary to reduced inositol 1,4,5-triphosphate generation and intracellular Ca^2+ ^mobilisation. Since we had not found any effect of IL-10 on TRAF6 or c-Fos, we wanted to see if we could find any evidence that IL-10 is acting through its effects on Ca^2+ ^mobilisation, and this was done using the PKC agonist PMA. Cells were treated with RANKL, RANKL plus IL-10, and RANKL plus IL-10 plus PMA, and viewed under the microscope. The suppressive effect of IL-10 was abolished by PMA treatment, as determined by extent of TRAP staining (Figure 4). Quantitative PCR results were in agreement, with PMA reversing the inhibitory effect of IL-10 on NFATc1 expression (Table 1).

**Table 1:** Effect of PMA on IL-10 induced suppression of NFATc1

Group	NFATc1 copies per 10^6 ^β-actin ± SEM	Relative change in expression compared to RANKL group
CONTROL	8.5 × 10^3 ^± 3 × 10^3 ^*	0.006 *
RANKL	1.4 × 10^6 ^± 5 × 10^5^	0
RANKL + IL-10	4.6 × 10^4 ^± 1 × 10^4 ^*	0.032 *
RANKL + IL-10 + PMA	7.9 × 10^5 ^± 9 × 10^4^	0.56

Quantitative RT-PCR analysis of NFATc1 mRNA expression in RAW264.7 cells. Cultures were treated with RANKL, RANKL and IL-10 or RANKL, IL-10 and PMA for 24 hours and total RNA extracted. Quantitative PCR was performed on a Techne Quantica PCR machine using the DNA binding dye SYBR-green and specific primers for murine NFATc1 and β-actin. For each sample the NFATc1 copy number was expressed as the relative copy number normalised to 10^6 ^β-actin copies. Samples were analysed in triplicate and are expressed as the mean of three separate experiments. * p < 0.05 versus RANKL group.

## Discussion

IL-10 is an anti-inflammatory cytokine that suppresses both immunoproliferative and inflammatory responses. It is produced primarily from T cells and activated macrophages. Animal studies implicate IL-10 in the development and progression of arthritis and chronic colitis. IL-10 has a critical role in the in vivo regulation of pro-inflammatory cytokine levels. It functions in a negative feedback loop, in which it suppresses the release of inflammatory cytokines and dampens the acute inflammatory response. Clinical studies and IL-10-deficient mice back up the theoretical importance of this work, as discussed in the introduction. IL-10 has been known for some time to have a potent inhibitory effect on osteoclastogenesis [18]. It prevents differentiation of osteoclast progenitors to preosteoclasts, however its mechanism of action is still unclear.

We have studied the expression of eleven osteoclast signalling genes and have found that one of the ways in which IL-10 directly inhibits osteoclastogenesis is by disrupting NFATc1 mRNA expression and nuclear translocation. NFATc1 is crucial for osteoclastogenesis, and so its downregulation may be expected to have major downstream effects. We also provide evidence that this disruption to NFATc1 may be mediated through the effect of IL-10 on Ca^2+ ^mobilisation. In addition, it was found that only NFATc1 isoform 1 transcribed from promoter P1 was downregulated by IL-10.

Osteoclast formation was not abolished in the presence of IL-10 and similarly NFATc1 levels did not revert to basal level in the IL-10 treated cells. Several pathways contribute to induction of NFATc1 levels and it may well be the case that IL-10 does not inhibit all of them. The initial expression of NFATc1 is dependent on both the TRAF6 and c-Fos pathways, which are unaffected by IL-10. RANKL stimulation also results in the induction of Ca^2+ ^oscillations which activate NFATc1 via a calcineurin-dependent mechanism, targeting it to the nucleus [2]. For the generation of osteoclasts it has been demonstrated that the high NFATc1 levels generated by autoregulation are necessary for cell lineage commitment [reviewed in [31]]. Therefore the reduction in levels, rather than complete abrogation, brought about by IL-10 will be sufficient to cause the potent inhibition of osteoclastogenesis shown in these studies.

There are two variants of NFATc1 in *Mus musculus *but only one of these, isoform1, was downregulated by IL-10. It is not known why the shorter isoform 2 is not similarly modified by IL-10. This may in part be related to the relative inability of RANKL to induce the expression of isoform 2 in RAW cells, or it could be that the conformation of the DNA is such that IL-10-induced transcription factors only have access to the P1 promoter. Linked in with this, it is likely that the finding that NFATc1 expression was not completely abolished is due to a combination of residual expression from the P1 promoter and the inability of IL-10 induced factors to suppress basal expression from the P2 promoter. Alternatively, it could also be that IL-10 acts only on a subset of the various pathways that contribute to NFATc1 upregulation.

The activity of NFATc1 is not only dependent on its level of expression, but also its location within the cell. It is active only when in the nucleus, as would be expected of a transcription factor. This nuclear transport relies upon active calcineurin which triggers dephosphorylation of NFATc1. Therefore we used antibodies to establish the intracellular distribution of NFATc1. The fact that very little staining, and in particular nuclear staining, was observed in the IL-10 sample strengthens our evidence that the inhibitory effect of IL-10 on osteoclastogenesis can be explained at least in part by its effect on NFATc1 expression and activity.

PKC plays a critical role in T cell receptor-induced NFAT activation [30]. Deficiency of PKC primarily abrogates NFAT transactivation. This NFAT transactivation defect appears to be secondary to reduced inositol 1,4,5-triphosphate generation and intracellular Ca^2+ ^mobilisation. Therefore, we set out to test whether IL-10 is disrupting NFATc1 activity through its effect on Ca2+ mobilisation. We found that PMA inhibits the suppressive effect of IL-10 on osteoclast formation, as determined by TRAP staining and NFATc1 expression. PMA is an activator of PKC, and so if PMA is overcoming the suppressive effects of IL-10, knowing how PMA acts it would appear that IL-10 is disrupting NFATc1 activity through its effect on Ca^2+ ^mobilisation. Therefore the effect of IL-10 could involve PKC as PMA reverses the suppressive action of IL-10.

The mechanism by which IL-10 interferes with osteoclast formation appears to be different from that used by other inhibitors, namely IFN-γ, IL-4, and TRAIL. IL-4 also downregulates NFATc1 but it acts through suppression of c-Fos [32], we did not find any evidence for c-Fos being downregulated although it remains possible that it could be inactivated by other means. IFN-γ causes rapid degradation of TRAF6 protein; we found no difference in TRAF6 mRNA levels but did not look at protein levels. Likewise, TRAIL interferes with the p38 pathway and there was no downregulation of p38 mRNA in IL-10 treated cultures.

It is valuable to pinpoint the effect of IL-10 on osteoclast signalling pathways since evidence is accumulating, particularly from studies of IL-10-deficient mice, to illustrate the importance of this cytokine in bone homeostasis. We have demonstrated that one of the ways by which IL-10 directly inhibits osteoclast formation is to suppress NFATc1 expression and activation. In vivo, IL-10 may be expected to have an even more pronounced effect due to its indirect action of suppressing RANKL expression [19] which would further reduce NFATc1 activation through TRAF6. This is the first report indicating that one of the ways in which IL-10 inhibits osteoclastogenesis is by suppressing NFATc1 activity.

## Conclusion

IL-10 acts directly on mononuclear precursors to inhibit NFATc1 expression and nuclear translocation, and we provide evidence that the mechanism may involve disruption of Ca^2+ ^mobilisation. We detected downregulation only of the NFATc1 isoform transcribed from promoter P1 and beginning with exon 1. This is the first report indicating that one of the ways in which IL-10 directly inhibits osteoclastogenesis is by suppressing NFATc1 activity.

## Methods

### Cell culture and cytokines

RAW264.7 cells were maintained in Dulbecco's modified Eagle's medium with 10% heat-inactivated fetal calf serum. They were transferred to a 24-well plate for cytokine addition and plated at a concentration of 10^5 ^cells per well. Cultures were fed every 2–3 days. TRAP staining (see below) was used to determine the extent of osteoclast formation. Cytokines were obtained from Insight Biotechnology (Wembley, UK). RANKL was added to cells to give a final concentration of 50 ng/ml, IL-10 was added to give a final concentration of 50–100 ng/ml.

### TRAP cytochemistry

Osteoclast formation was evaluated by quantification of TRAP-positive cell number using a modification of the method of Burstone [33]. After incubation, coverslips were washed in phosphate-buffered saline, fixed in 10% formalin for 10 min, and stained for acid phosphatase in the presence of 0.05 M sodium tartrate (Sigma). The substrate used was naphthol AS-BI phosphate (Sigma). The number of TRAP-positive mono and multi nuclear cells was counted using an eyepiece graticule at a magnification of ×100.

### Selection of genes for study

NFATc1, c-jun [34], RANK [35], TRAF6 [36], p38, NF-κB, and c-Fos [37] are well known to play an important role in osteoclastogenesis. It has recently been shown that signals other than those transmitted by RANKL and M-CSF are required for osteoclastogenesis, and DAP12 [38] and FcγRIIB [39] are key molecules in this respect. Gab2 is a novel downstream effector of RANKL signalling [40]. Pim-1 was chosen because it interacts directly with human NFATc1 [41], and it was thought that it may play a role in the mouse signalling pathway.

### Extraction of RNA and quantitative RT-PCR

RNA was extracted using a Qiagen kit from control, RANKL treated, and RANKL plus IL-10 treated cells that had been incubated for 48 hours. The RNA was reverse transcribed using a Sigma kit. Primers were designed using Primer3 and their sequences are given below. Real-time PCR was conducted with a Techne Quantica machine and Quansoft software using the DNA-binding dye SYBR Green (Sigma) for the detection of PCR products. For the generation of standard curves, the corresponding cDNA was cloned into pGEM-T Easy (Promega). The concentration of DNA plasmid stock was determined by the OD at 260 nm and agarose gel electrophoresis. Copy number for each plasmid was calculated on these measurements. The linear range of the assay was determined by the amplification of log serial dilutions of plasmids from 500 to 5 × 10^6^. The progress of the PCR amplification was monitored by real-time fluorescence emitted from SYBR Green during the extension time. The cycles were 95°C for 15 min, followed by 35 cycles of 95°C for 20 s, 58°C for 20 s, and 72°C for 20 s. At the end of each PCR run, a melt curve analysis was performed to show the absence of non-specific bands. For each sample, mRNA levels were expressed as relative copy number normalised against β-actin mRNA. This was achieved by constructing a standard curve for each PCR run from serial dilutions of purified plasmid DNA with specified amplicon. The mRNA copy number was calculated for each sample from the standard curves by the instrument's software. Samples were analysed in triplicate. For each sample copy number relative to 10^6 ^β-actin copies in the same sample was calculated.

Primers used for PCR were as follows: NFATc1 sense, 5'-CCGTTGCTTCCAGAAAATAACA-3'; NFATc1 antisense, 5'-TGTGGGATGTGAACTCGGAA-3'; NFATc1 P1 isoform sense, 5'-GCCAAGTACCAGCTTTCCAG-3'; NFATc1 P1 isoform antisense, 5'-AGGGTCGAGGTGACACTAGG-3'; NFATc1 P2 isoform sense, 5'-TTCGATTTCCTCTTCGAGTTC-3'; NFATc1 P2 isoform antisense, 5'-TGATTGGCTGAAGGAACAGC-3'; β-actin sense, 5'-GTCATCACTATTGGCAACGAG-3'; β-actin antisense, 5'-CCTGTCAGCAATGCCTGGTACAT-3', which yield products of 152, 185, 165, and 197 bp, respectively.

### Immunofluorescence

Cells were grown on 2 mm glass coverslips in 6 well plates for 48 hours with or without RANKL or RANKL plus IL-10. The coverslips were then removed, washed in PBS, fixed in 4% paraformaldehyde/phosphate-buffered saline pH 7.2 for 15 minutes, washed in 70% alcohol and then air dried. The cellular distribution of NFATc1 was assessed as follows. Cells were rehydrated through graded alcohols, permeablised with 0.1% Triton X-100, incubated with 1% goat serum for 30 minutes to block non-specific binding and then incubated with a specific mouse NFATc1 monoclonal (Santa Cruz Biotechnology, Heidelberg, Germany) diluted 1:50 in 1% goat serum for 1 hour. The cells were washed in PBS, incubated for 2 hours with a biotinylated goat anti-mouse secondary (Vector Labs, Burlingame, USA), washed in PBS and then incubated for 2 hours with fluorescein conjugated streptavidin (Vector Labs, Burlingame, USA) Immunofluorescence was then visualised using a Leica HC microscope. Photographs were taken with a JVC TK C13603 digital camera linked to image pro plus software at a magnification of ×400 or ×1000.

### Statistical analysis

Differences between groups were assessed using Fisher's analysis of variance (StatView version 5.01, Abacus Concepts, Berkley, CA). A difference of p < 0.05 was considered significant.

### Bioinformatics analysis

Information, including sequence data, on the genes discussed in this paper was obtained via the NCBI website [42].

## Abbreviations

IFN, interferon

IL, interleukin

M-CSF, macrophage colony stimulating factor

NFAT, nuclear factor of activated T cells

PKC, protein kinase C

PMA, phorbol myristate acetate

RANK, receptor activator of NF-κB

RANKL, receptor activator of NF-κB ligand

TNF, tumour necrosis factor

TRAF, TNF receptor associated factor

TRAIL, TNF-related apoptosis-inducing ligand

TRAP, tartrate-resistant acid phosphatase

## Authors' contributions

KE participated in the selection of genes for study, carried out the molecular genetic work, and drafted the manuscript. SF conceived of and directed the study, carried out the TRAP cytochemistry and immunofluorescence work, and helped to draft the manuscript. Both authors read and approved the final manuscript.
